# Physical Properties of Additively Manufactured Tooth-Colored Material Attached to Denture Base-Colored Material in a Printed Monolithic Unit

**DOI:** 10.3390/polym15092134

**Published:** 2023-04-29

**Authors:** Amr Mohamed, Atsushi Takaichi, Yuka Kajima, Hidekazu Takahashi, Noriyuki Wakabayashi

**Affiliations:** 1 Advanced Prosthodontics, Graduate School of Medical and Dental Sciences, Tokyo Medical and Dental University, 1-5-45 Yushima, Bunkyo-Ku, Tokyo 113-8549, Japan; amr.gamal.rpro@tmd.ac.jp (A.M.); y.kajima.rpro@gmail.com (Y.K.); wakabayashi.rpro@tmd.ac.jp (N.W.); 2 Course for Oral Health Engineering, Faculty of Dentistry, Tokyo Medical and Dental University, 1-5-45 Yushima, Bunkyo-Ku, Tokyo 113-8549, Japan; takahashi.bmoe@tmd.ac.jp

**Keywords:** additive manufacturing, monolithic, PMMA denture base, water sorption, water solubility, bond strength, CAD/CAM, material jetting, polyjet printing, 3D printing

## Abstract

Additive manufacturing is an emerging technology that has been successfully used in dentistry for denture fabrication. However, the conventional issue of tooth debonding exists in additively manufactured dentures. In this study, we investigated the physical properties of conventional teeth attached to a heat-cured denture base material compared to additively manufactured tooth-coloured materials attached to denture base-coloured materials in a printed monolithic unit. We designed a model consisting of a tooth attached to a cylindrical base to fabricate the additively manufactured group and the conventional group. All groups were tested for fracture load before and after thermocycling, water sorption, solubility, and shape accuracy. The Mann–Whitney U test was used for statistical analysis. The fracture load of the additively manufactured group was significantly higher than that of the conventional group after thermocycling (*p* = 0.019). The water sorption of the conventional group was significantly lower than that of the additively manufactured group (*p* = 0.000), whereas there was no significant difference in the water solubility between them (*p* = 0.192). The shape accuracy of the additively manufactured group was significantly better than that of the conventional group (*p* < 0.05). In conclusion, additive monolithic manufacturing technology may provide an alternative way to enhance the fracture load between the teeth and denture bases.

## 1. Introduction

Over recent decades, complete dentures have become the first affordable choice for rehabilitation of edentulous patients [1,2]. Polymethyl methacrylate (PMMA) is generally used for their fabrication because of several advantages, including its biocompatibility, light weight, pleasant aesthetic quality, low cost, and simple processing and repair techniques [3]. However, PMMA has poor mechanical properties and undergoes dimensional changes [2,4]. Despite the wide popularity of PMMA denture fabrication, the traditional fabrication technique still has some disadvantages, such as the long time required for multiple appointments, human-induced errors, and laboratory costs [5]. Recently, digital dentistry has paved the way for new fabrication techniques using computer-aided design and manufacturing (CAD/CAM). It is possible to fabricate dentures using either milling or additive manufacturing techniques [6]. In the milling technique, pre-polymerised disks are milled to produce dentures. However, in the printing technique, 3D parts are built in the form of consecutive layers above each other [5]. This printing technique represents a great leap in manufacturing technology because it can be used to fabricate more complex shapes, saves time, is less expensive, and produces less material waste compared to the milling and traditional techniques, especially in patch production [5].

The detachment of teeth from PMMA denture bases has been considered to represent 26–30% of denture repair scenarios [7,8]. Although artificial teeth can chemically bond to denture base-resin material, the types of artificial teeth and denture base-resin materials, the polymerisation method, and the sensitivity of the processing technique might negatively affect the bond strength, as happens in the case of incomplete removal of wax remnants from the ridge lap surface of artificial teeth and the unintentional application of a separating medium on the bonding interface in traditional fabrication techniques [9,10,11,12,13]. In digitally fabricated dentures, the teeth and denture bases are usually fabricated separately (either by milling or additive manufacturing) and bonded together; however, debonding still occurs [14].

To address the crucial issue of the detachment of artificial teeth from denture bases in complete and partial dentures, numerous studies have thoroughly investigated different techniques to improve bond strength. Klaiber et al. reported a remarkable enhancement in the shear bond strength between custom prefabricated teeth and milled denture bases after monomer application [15]. Alharbi et al. reported a higher fracture load between 3D printed denture teeth and printed base-resin material than between artificial teeth and conventional heat-cured denture base resin [16].

Another critical issue affecting acrylic resins is their tendency to absorb water (water sorption) and their water solubility. Both properties are important because they directly affect the strength of and the dimensional changes in acrylic resins in the patient’s mouth [17,18]. Therefore, it is very important to minimize these properties to prevent the possible formation of cracks that might jeopardize the strength and longevity of the denture bases [19].

Maintaining the shape accuracy of fabricated dentures following the design model is another concern. To ensure maximum fit and performance, the fabricated dentures should maintain the exact dimensions of the design model; otherwise, retention and stability will be negatively affected. Unfortunately, conventional dentures fabricated with traditional processing techniques, including wax elimination and packing of acrylic resin, may be subject to dimensional changes due to polymerization shrinkage [20]. CAD/CAM dentures show greater dimensional accuracy than conventionally fabricated dentures [21].

Recently, additive monolithic manufacturing using material jetting technology has been introduced, with which different materials with different colours and properties can be printed together in a monolithic (single) unit [22]. This technology works through the extrusion of an ultraviolet (UV)-curable photopolymer liquid in the form of layers, using different nozzles for different materials and colours simultaneously, which are subsequently cured using the UV instantly [22]. Although material jetting technology can potentially produce highly aesthetic dentures, the physical properties, such as bonding strength, water sorption, solubility, and shape accuracy, which are important properties for denture fabrication, have yet to be evaluated.

Therefore, this study aimed to investigate the fracture load between additively manufactured tooth-coloured material and denture base-coloured material in a monolithic printed unit compared to that between conventional prefabricated teeth and conventional heat-cured denture base resin before and after thermocycling. In addition, water sorption, water solubility, and shape accuracy were investigated. The null hypothesis was that the fracture load, water sorption, water solubility, and shape accuracy would not be affected by the fabrication techniques.

## 2. Materials and Methods

### 2.1. Digital Data Preparation

**Figure 1:**
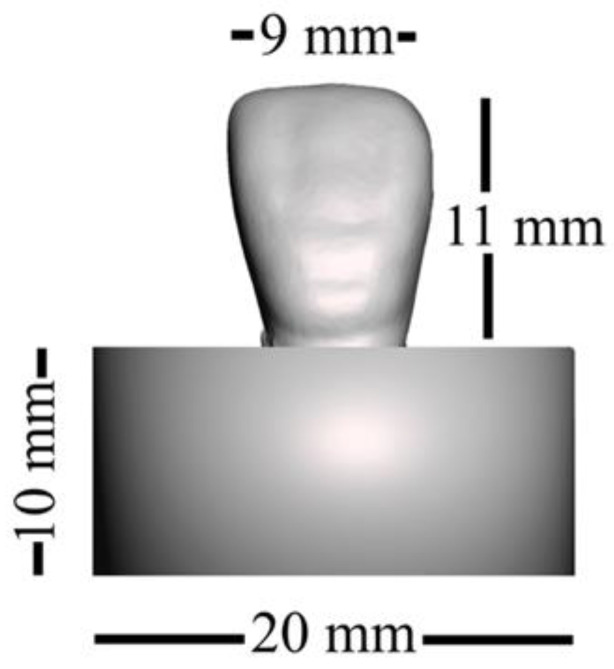
Dimensions of the designed specimen.

**Figure 2:**
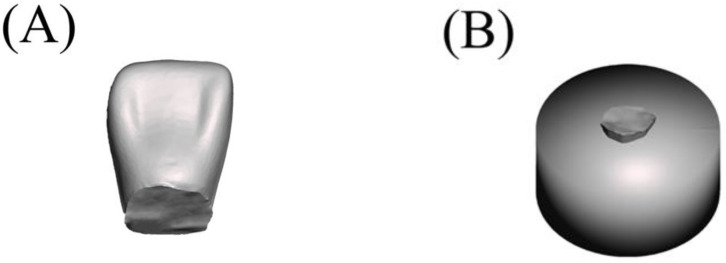
Split parts of the designed specimen: (**A**) tooth, (**B**) cylindrical base with collar part.

We prepared 40 specimens for the fracture load test, which followed the ISO/TS 19736 standard with some modifications [23,24,25]. The sample size was calculated to provide 5% alpha error and 80% power using statistical power analysis software (G*Power 3.1.9.3 for Mac OS X). We created an STL file by scanning an artificial maxillary right central incisor and exporting the data (Endura Anterio HC5, Shofu Inc., Kyoto, Japan). Subsequently, the fitting surface of the neck of the tooth was marked and extruded for 3 mm along the long axis using 3D modelling software (Autodesk Meshmixer 3.5, Autodesk, San Rafael, CA, USA). Next, the extruded part was cut perpendicular to the long axis of the tooth using the same software. We then used CAD software (Sharp 3D, Shapr3D Zrt., Budapest, Hungary) to merge the STL file with a created cylinder of dimensions ø20 × 10 mm, as shown in Figure 1. The planned model was split into a cylindrical base and tooth parts at the connecting interface (Figure 2).

#### 2.1.1. Fabrication of the Additively Manufactured Group

**Figure 3:**
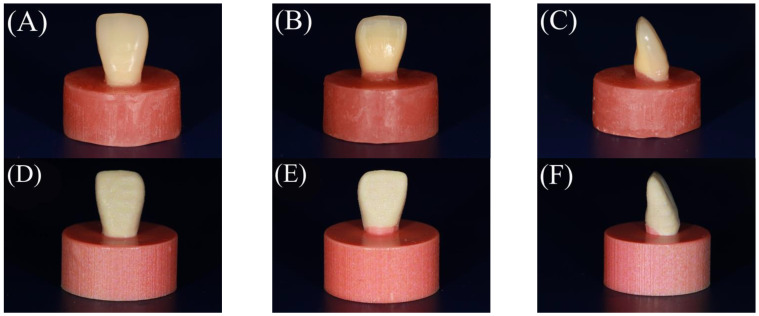
Buccal, palatal, and side views of the finished specimens of the tested groups. (**A**–**C**) The conventional group and (**D**–**F**) the additively manufactured group.

A 3D printer (Polyjet J55 prime, Stratasys, Edina, MN, USA) was employed to fabricate 20 specimens using RGD851 VeroMagentaV material with a shade (R255, G136, B136, A1) for the gingival part and RGD835 VeroUltraWhite material with a shade (R240, G222, B186) for the tooth part. The chemical compositions are presented in Table 1. The cylindrical base units faced the platform in a perpendicular direction during the printing process. The “High-Quality, High-Speed (HQHS)” mode was adopted. The layer thickness was set to 18.75 μm. The teeth and denture base parts were printed in the form of successive layers of the previously mentioned materials on top of each other in a monolithic pattern (single unit). Each printed layer was cured using ultraviolet (UV). Subsequently, the specimens were removed and finished (Figure 3D–F).

#### 2.1.2. Fabrication of Conventional Group

A conventional compression moulding technique was used to fabricate 20 specimens. Initially, one of the additively manufactured specimens was used to create a silicone mould with a silicone material (Duplicone, Shofu Inc., Kyoto, Japan). Subsequently, the maxillary right central incisor (Endura Anterio HC5, Shofu Inc., Kyoto, Japan) was placed in the corresponding space within the silicone mould. The silicone mould was then filled with molten baseplate wax. A tooth attached to the cylindrical wax part was obtained. A thin coating of the separating medium was then applied to the inner surfaces of the dental flasks. A type 2 plaster (ADVASTONE, GC, Tokyo, Japan) was used to flask wax specimens. Following the removal of the wax, the powder and liquid (Acron, GC, Tokyo, Japan) from the heat-cured acrylic resin were mixed and packed (the chemical composition is presented in Table 1) according to the manufacturer’s instructions, and a long polymerization cycle was applied at 73 °C for 10 h in hot water. Subsequently, the specimens were finished (Figure 3A–C). Before testing, the specimens were maintained in distilled water at room temperature for 24 h.

### 2.2. Thermocycling of Specimens

Thermocycling was performed for half of the specimens (n = 10) of each group with 10,000 cycles in distilled water between 5 °C and 55 °C and a 30 s dwell time using a thermal cycling machine (Thermal Cycling K178, Tokyo Giken Inc., Tokyo, Japan).

### 2.3. Fracture Load Test

**Figure 4:**
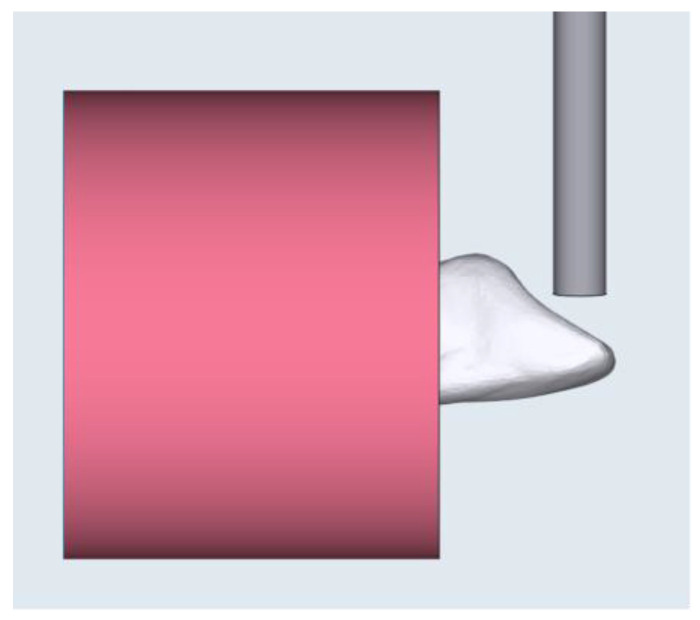
Illustration of the direction of the load applied on the tooth surface.

The fracture load was investigated using a universal testing machine (AG-X, Shimadzu Corp., Kyoto, Japan) with a load of 500 N, utilizing a 4 mm diameter flat shear-pin at 90° to the palatal surface of the teeth with a crosshead speed of 1 mm/min until fracture, as shown in Figure 4. To ensure that all mounted specimens had the same orientation as the load direction, a specially designed silicone index was created. The fracture load was recorded in Newtons (N).

### 2.4. Identification of the Mode of Failure

A digital microscope (VHX-S50, Keyence, Osaka, Japan) was used to determine the failure mode. The mode of failure was determined as one of the following: cohesive failure (if the fracture path was entirely contained within either the denture base resin or the tooth), adhesive failure (if the fracture path was at the interface between the denture base resin and the tooth), and mixed failure (if the fracture path involved both adhesive and cohesive failures).

### 2.5. SEM Analysis

For further detailed investigation of the fracture surface, a scanning electron microscope (JSM-7900F, JEOL, Tokyo, Japan) with an accelerating voltage of 5 kV was used. Before carrying out the SEM investigation, the samples were sputtered under a vacuum with a thin layer of gold (about 10 nm), which is a highly conductive material, to obtain high-quality images for the non-conductive samples.

### 2.6. Water Sorption and Solubility Tests

The ISO 20795-1:2013 standard was adopted to determine the amount of water sorption and the solubility of the specimens, except with regard to the specimen dimensions. Specimens with dimensions of ∅ 20 mm × 0.5 mm thickness were prepared from the denture base parts of the previously fabricated specimens used for the fracture load test. The volume (V) of each disc specimen was determined using the following equation: V = πr^2^ × h, where (r) is the radius of the disc (mm), and (h) is the thickness in mm. The diameter was determined as the average of the diameters measured at three different locations, whereas the thickness was determined as the average of thicknesses measured at five different points using a digital calliper (MDC-25M; Mitutoyo, Japan). The disc specimens were then stored in an auto dry desiccator (As One, Tokyo, Japan) at room temperature (24 ± 1 °C) for 24 h, for which a solid high-polymer electrolyte membrane and H_2_O electrolytic discharge system were used. Subsequently, the specimens were weighed using an analytical balance (HR-100AZ; A & D, Tokyo, Japan) after the mass became constant within 0.2 mg, and this mass was designated M1. The specimens were immersed in a water bath at 37 ± 1 °C for one week in a thermal cycling machine (Thermal Cycling K178; Tokyo Giken Inc., Japan). They were then wiped with absorbent paper and weighed for 60 s, and the mass was designated M2. The disc specimens were stored in an incubator (As One, Tokyo, Japan) at 37 ± 1 °C for 24 h, for which a Peltier element under a forced convection system was used, and then the disc specimens were weighed and the mass was designated M3.

The water sorption was then calculated using the following equation: water sorption (μg/mm^3^) = M2 − M3/V, where M2 is the mass of the disc specimen after immersion in water (μg), M3 is the reconditioned mass of the disc specimen after immersion in water (μg), and V is the volume of the disc specimen in mm^3^. The water solubility was calculated using the following equation: water solubility = M1 − M3/V, where M1 is the conditioned mass of the disc specimen before immersion in water (μg), M3 is the reconditioned mass of the disc specimen after immersion in water (μg), and V is the volume of the disc specimen (mm^3^).

### 2.7. Shape Accuracy

The accuracy of the different fabrication techniques used in this study was investigated in terms of trueness by comparing the shapes of the obtained specimens to the reference design model, using superimposition to detect the distance between the two surfaces. All specimens were scanned using a lab scanner (3D Edge Scanner, DOF, Seoul, Republic of Korea) and the STL files were exported. Subsequently, the STL data for each specimen were initially superimposed over the reference design model using the “initial alignment” option and then the “best-fit” option, which is based on the minimal distance alignment between two surfaces, in 3D modelling software (Geomagic Control 2018, 3D Systems, Rock Hill, SC, USA). The reported root-mean-square (RMS) value from the superimposition analysis for each group was used for comparison. Furthermore, the “3D compare” option in the same 3D modelling software was used to demonstrate the direction of the deviation in each group from the reference design model in a colour-coded map.

### 2.8. Statistical Analysis

The Statistical Package for the Social Sciences (SPSS) software was used for statistical analysis (IBM SPSS Statistics for Mac. V25; IBM Corp., Armonk, NY, USA). The Shapiro–Wilk test was used to check the normality of the data distribution. If the data showed a normal distribution, the independent-samples t-test was used. However, if the data showed no normal distribution, the independent-samples Mann–Whitney U test was used (*p* = 0.05).

## 3. Results

### 3.1. Fracture Load

Only the data for the thermocycled conventional group did not show a normal distribution (*p* = 0.005, *p* < 0.05); therefore, the independent-samples Mann–Whitney U test was used. The medians of the fracture loads are presented in Table 2. The fracture load of the conventional group was significantly higher than that of the additively manufactured group (*p* = 0.005) before thermocycling. However, the fracture load of the additively manufactured group was significantly higher than that of the conventional group (*p* = 0.019) after thermocycling. The fracture load of the conventional group was significantly reduced after thermocycling, whereas that of the additively manufactured group was not significantly affected by the thermocycling process.

### 3.2. Mode of Failure

**Figure 5:**
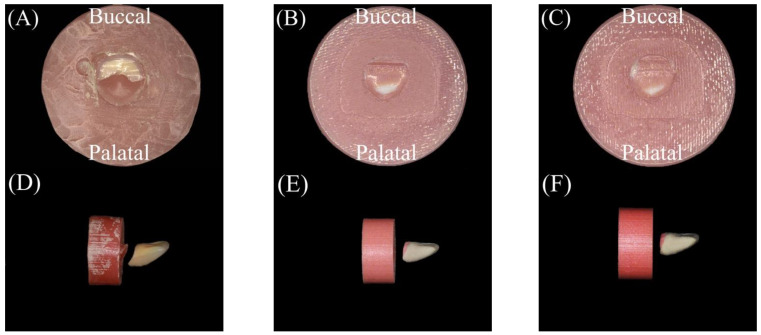
Illustration of the orientation of fracture lines and the modes of failure: (**A**,**D**) conventional group (mixed), (**B**,**E**) additively manufactured group (cohesive), and (**C**,**F**) additively manufactured group (mixed).

Figure 5 shows the fracture patterns for the conventional and additively manufactured groups. Table 3 shows the numbers of samples with the different modes of failure before and after thermocycling in both groups. Table 4 summarises the percentages for the various modes of failure within both groups.

**Figure 6:**
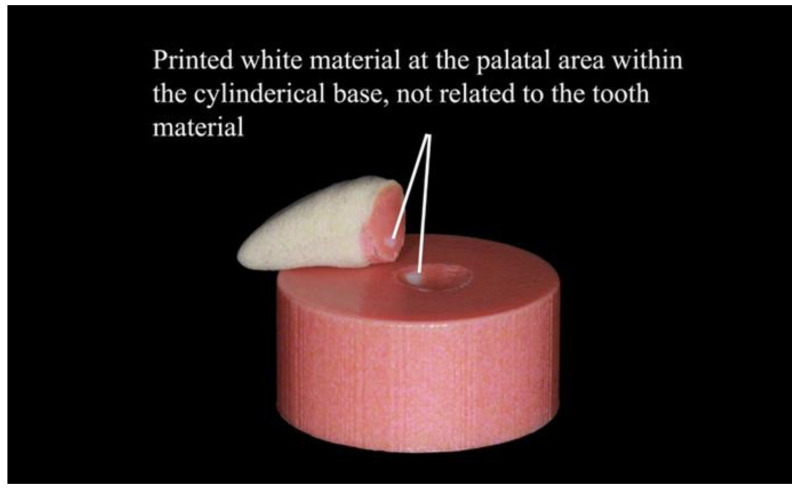
Demonstration of the printed white material within the cylindrical base.

Note that the white material appearing in the palatal areas of the additively manufactured specimens in Figure 6 was not related to the tooth material. It was printed within the cylindrical base for technical reasons following the manufacturer’s instructions. Therefore, it was not considered in the identification of the failure modes.

### 3.3. SEM Analysis

**Figure 7:**
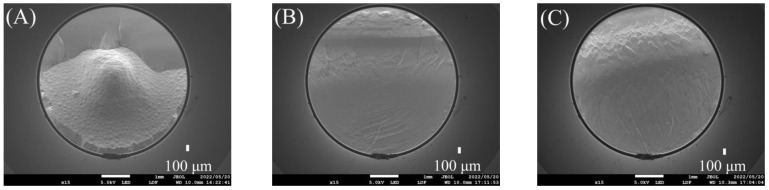
The modes of failure under SEM: (**A**) conventional group (mixed), (**B**) additively manufactured group (cohesive), and (**C**) additively manufactured group (mixed).

**Figure 8:**
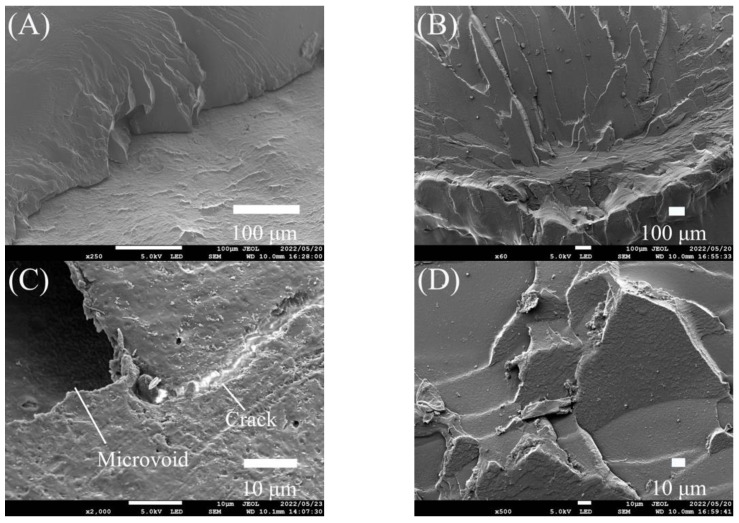
SEM analysis of the fractured surfaces of the tested groups: (**A**,**C**) conventional group and (**B**,**D**) additively manufactured group.

Further investigation of the fractured surfaces was performed, and the failure modes were confirmed using SEM (Figure 7). A brittle fracture pattern with multilevel fractures and cleavage facets was detected in the conventional group, as shown in Figure 8A. As seen in Figure 8C, the cracks at the fracture site were found to originate from a significant microvoid, whereas the additively manufactured group displayed brittle fracture behaviour, as illustrated in Figure 8B. Mirror surfaces were also detected, as shown in Figure 8D.

### 3.4. Water Sorption and Solubility

The mean and standard deviations for both groups from the water sorption and solubility tests are presented in Table 5. The water sorption test data showed a normal distribution (*p* > 0.05). Therefore, an independent-samples t-test was used. The mean water sorption value for the conventional group was significantly lower than that for the additively manufactured group (*p* = 0.000). The data for the water solubility test did not show a normal distribution (*p* < 0.05). Therefore, we used the independent-samples Mann–Whitney U test. There was no significant difference in the water solubility between the groups (*p* = 0.192).

### 3.5. Shape Accuracy

**Figure 9:**
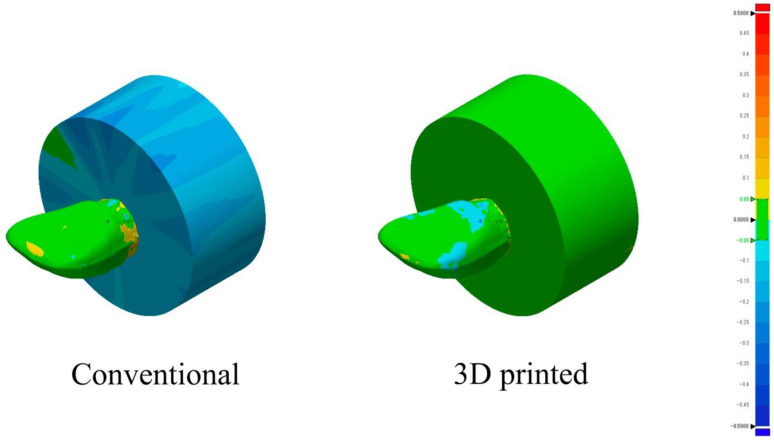
Three-dimensional (3D) comparison of the superimposed groups and the reference design model using a colour-coded map to illustrate the direction of deviation.

According to the Shapiro–Wilk test, the data for both groups showed normal distributions (*p* > 0.05). The mean and standard deviation values for the groups are presented in Table 6. The mean RMS value for the additively manufactured group was significantly lower than that for the conventional group (*p* < 0.05). The directions of deviation from the reference design model for both groups are illustrated in Figure 9, where the green, blue, and red zones indicate the minimum deviation (within the tolerance range), inward deviation, and outward deviation, respectively. An apparent inward deviation was observed for the denture base in the conventional group. In contrast, the denture base part in the additively manufactured group showed good shape accuracy, as indicated by the green colour.

## 4. Discussion

This study investigated the fracture load, water sorption, solubility, and shape accuracy of conventional compression moulding compared to additive monolithic manufacturing fabrication techniques. The null hypothesis was partially rejected because there were significant differences between the two fabrication techniques in terms of the fracture load, water sorption, and shape accuracy. Thermocycling had no significant effect on the fracture load in the additively manufactured group, and there was no significant difference in the water solubility between the two fabrication techniques.

In the present study, it was necessary to fabricate a silicone index to obtain a standardised 3D position for all the test specimens in the direction of the applied load. This is because the resultant load will vary if the inclined palatal surfaces of the test specimens are rotated, and thus the relationship to the testing load direction would be altered, resulting in different force resolutions [26].

To imitate the thermal stress caused by temperature changes in the oral cavity and investigate their potential effect on the fracture load between denture teeth and denture base materials, we thermocycled test specimens for 10,000 cycles in distilled water with a temperature range of 5–55 °C [24,27].

Before thermocycling, the conventional group had a significantly higher fracture load than the additively manufactured group. The reason behind this result was most likely the difference in the physical properties of the materials used in the two fabrication techniques, as the flexural strength of the additively manufactured teeth and denture base-shade materials (75–110 MPa) was lower than that of the artificial teeth and heat-cured denture base-resin materials used in the conventional group (143 and 118 MPa, respectively) [28,29,30,31]. After thermocycling, the fracture load of the conventional group was significantly lower than that of the additively manufactured group. This was believed to be due to the difference in the thermal expansion coefficients of the denture base and artificial teeth materials used in the conventional group, which led to cyclic stress at the bonding interface due to the temperature changes during the thermocycling procedure [32,33]. As a result, fatigue was generated within those materials, which would have facilitated fracture initiation and debonding across the bonding interface [32]. However, there was no significant change in the fracture load of the additively manufactured group after thermocycling, which might have been due to the fact that the denture base and teeth materials had the same physical properties, including the thermal expansion coefficient, which minimized the thermal stress generated between them at the bonding interface.

Both groups satisfied the ISO 19736 standard (adhesive failure less than 33%, as defined by the ISO standard) [34]. The conventional group’s fracture load demonstrated 5% adhesive mode of failure after thermocycling, which was believed to be because of the difference in the thermal expansion coefficient, as mentioned before. However, the conventional group showed 95% mixed mode of failure, which could be attributable to the cracks initiated by voids identified at the bonding site during the load application. These flaws might have occurred in the processing or packing steps because of air entrapment or the existence of residual monomers. The additively manufactured group demonstrated 20% cohesive mode of failure (including thermocycled and non-thermocycled specimens) within the denture base resin. This could be attributable to the formation of strong interfacial adhesion resulting from the distinctive fabrication technique, in which different materials are printed in a single body, eradicating the need for a bonding step [35]. This is in agreement with Liu et al., who tested the tensile strength at the interface between different printed materials using polyjet technology and concluded that there was no interfacial failure between them [22]. In contrast, the rest of the specimens from the additively manufactured group demonstrated 80% mixed mode of failure (including thermocycled and non-thermocycled specimens). These failures could return to defects as porosities among the printed tracks or layers that could be introduced during the printing process, which, in turn, helped in crack propagation upon application of the load during the testing procedure. In addition, micropores might have been formed during the testing procedure, as the load was applied in a direction parallel to that of the printed layers, which might have resulted in debonding of the layers and the formation of micropores in between them [36]. Thus, crack propagation might have resulted in a combination of adhesive and cohesive modes of failure (mixed mode of failure).

The water sorption of resin materials is primarily affected by their chemical composition and the presence of empty spaces within them [37]. Chemical composition plays an important role in the chemical interaction with water molecules through the presence of hydrophilic polar sites, as in the residual monomers that tend to interact with water molecules [37,38]. In the present study, the water sorption for the additively manufactured group was significantly higher than that for the conventional group, whereas there was no significant difference in water solubility between the two groups. This might be attributable to the existence of a lower amount of residual monomers within the conventional group owing to a higher degree of polymerization compared to the additively manufactured group [20]. This might have been because the conventional group was heat-cured for a long duration at a high temperature compared to the curing method used for the additively manufactured group, which utilized UV for a very short time, possibly resulting in a lower amount of residual monomers within the conventional group, as previously reported [20,39,40]. In addition, the differences in their original chemical compositions might have had a significant impact on their interactions with the water molecules [41,42].

According to the ISO 1567:1999(E) standard, water sorption should not exceed 32 μg/mm^3^ for all polymers, and water solubility should not exceed 1.6 μg/mm^3^ for all polymers except self-cure acrylic resin (8.0 μg/mm^3^) [43]. Therefore, the conventional group satisfied the ISO standard, unlike the additively manufactured group.

Shape accuracy was evaluated in terms of trueness, which is defined as “the closeness of agreement between the arithmetic mean of a large number of test results and the true or accepted reference value” according to the ISO 5725-1 standard [44]. The additively manufactured group showed a significantly lower deviation from the reference design model than the conventional group. This result might be attributable to the polymerization shrinkage that occurred during the processing of the heat-cured acrylic resin in the conventional group. This was confirmed from the results of the 3D comparison of the two techniques, which showed almost a whole blue colour for the processed cylindrical denture base part in the conventional group, indicating an inward deviation from the reference design model [45]. However, the additively manufactured group almost maintained the shape of the reference design model, including the tooth and denture base parts, as it nearly showed a whole green colour, indicating that it lay within the true value of the reference design model. This result contradicts previous reports that showed no significant difference in trueness between conventional and additively manufactured dentures, which might be attributable to the different additive manufacturing techniques used in the two studies; SLA printing technology was used in the reported study and material jetting technology was used in our study [46]. The additively manufactured group satisfied the ISO standard 20795-1:2008(E) in terms of shape capability. This study was limited in that only composite artificial teeth were used in the conventional group. In addition, a dynamic loading test using a chewing simulator was not conducted. Although versions of the additively manufactured materials dedicated to denture fabrication were not available on the market at the time when the present study was conducted, they are now. Further investigations are required using the versions of those additively manufactured materials dedicated to denture fabrication with material jetting technology.

## 5. Conclusions

Additive monolithic manufacturing using material jetting technology offers a promising way to eradicate the need for bonding between printed materials. The additively manufactured group showed a significantly better fracture load (median value = 83.99 N) than the conventional group (median value = 36.12 N) after thermocycling. In addition, only the fracture load in the conventional group significantly deteriorated after exposure to thermal stresses. According to ISO 19736, both fabrication techniques demonstrate clinically acceptable fracture loads. The additively manufactured group showed exceptional dimensional accuracy compared to the conventional group. Furthermore, the additively manufactured group did not satisfy the ISO 20795-1:2013 standard for water sorption.

## Figures and Tables

**Table 1:** Details of the materials used.

Brand Name.	Manufacturer	Chemical Composition	Processing Technique
**Acron**	GC, Tokyo, Japan	Powder: methacrylic acid ester polymer, others Liquid: methyl methacrylate, others	Heat polymerization
**RGD835 VeroWhite**	Stratasys, Edina, MN, USA	Exo-1,7,7 trimethylbicyclo [2.2.1] hept-2-yl acrylate 5888-33-5, tricyclodecane dimethanol diacrylate 42594-17-2, titanium dioxide 13463-67-7, others	Ultraviolet (UV) curing
**RGD851** **VeroMagenta**	Stratasys, Edina, MN, USA	Trialkyl(C = 1~3)bicarbomonocyclic acrylate, substituted alkenyl(C = 1~4)-morpholine, substituted alkano(C = 1~4)carb opolycyclic-diylbis(alkylene(C = 1 ~4)) bisacrylate, isoalkyl (C = 2~5) idenediphenol, oligomeric reaction products with substituted epoxyalkane(C = 2~5), esters with acrylic acid, acrylic acid, multifunctional polyether acrylic acid ester, others	UV curing

**Table 2:** Effect of thermocycling on the fracture loads for both fabrication techniques.

Fabrication Technique	Median of Fracture Loads (SD) (Newton)		
	Non-Thermocycled	Thermocycled	*p*-Value
**Conventional**	159.79 ^A^ (50.8)	36.12 ^1^ (36.5)	0.00
**Additive manufacturing**	91.75 ^B^ (13.9)	83.99 ^2^ (12.7)	0.24

Identical superscript letters or numbers in the same column denote that there was no significant difference (*p* > 0.05), and the *p*-value indicates the effect of thermocycling on each group.

**Table 3:** Quantitative assessment of different types of modes of failure following thermocycling in the conventional and additively manufactured groups.

	Fabrication Technique	Modes of Failure		
		Adhesive	Cohesive	Mixed
**Before** **Thermocycling**	Conventional	0	0	10
	Additive manufacturing	0	0	10
**After** **Thermocycling**	Conventional	1	0	9
	Additive manufacturing	0	4	6

**Table 4:** Analysis of the modes of failure in the conventional and additively manufactured groups.

Figure 5.	Modes of Failure		
	Adhesive	Cohesive	Mixed
**Conventional**	5%	0%	95%
**Additive manufacturing**	0%	20%	80%

**Table 5:** Means and standard deviations for the water sorption (Wsp) and solubility (Wsp) for both groups.

Fabrication Techniques	Wsp (μg/mm^3^)	Wsl (μg/mm^3^)
	Mean ± SD	
**Conventional**	22.7 ± 3.2 ^A^	0.96 ± 1.98^1^
**Additive manufacturing**	33.7 ± 5.0 ^B^	2.13 ± 2.91^1^

Identical superscript letters or numbers in the same column denote that there was no significant difference (*p* > 0.05).

**Table 6:** Mean root-mean-square (RMS) values and standard deviations from the best-fit test for both groups.

Fabrication Techniques	RMS (mm)	St. Deviation
**Conventional**	0.1227	0.04458
**Additive manufacturing**	0.0384	0.00575

## Data Availability

The data that support the findings of this study are available from the corresponding author, upon reasonable request.
